# Prison Administration and the Training of Prison Officials.—II

**Published:** 1898-03-19

**Authors:** 


### Modern Sociology.

### PRISON ADMINISTRATION AND THE TRAINING OF PRISON OFFICIALS.—II.

Before turning to the more theoretical aspects of the subject, it may be well simply to indicate the points on which the training of men differs from that of women. The schools for men are at present only two in number. That of Chelmsford Prison was begun in October, 1896, and is under the able direction of Major Darnell, who has had much experience in the prisons of Borstal, Knutsford, and Leicester. The Hull school is of more recent establishment, having only been begun early in the past year, but its superintendent, Mr. Chidley, has long done good service as a prison governor, and takes great interest in his new work. At the present time there are only about fifteen young men in each of these schools, who go through a course of four months' instruction in all departments of prison duty; it is to be hoped that this number will soon be largely augmented, and that increased accommodation for the reception of probationers will shortly be prepared. The practical work in which these aspirants are exercised is, to some extent, the same as that of which we have already given the details in regard to women probationers. As in their case, the men, under the guidance of an experienced warder, are instructed in the diverse treatment of convicted and unconvicted prisoners, the oversight of industrial work, the portioning out of food and clothing, the discipline in and out of the cells, the hearing and reporting of complaints or breaches of rule, with all the different functions which pertain to them in the bakery, the kitchen, and garden grounds. Hospital duty is a separate subject, of which we shall have to write in fuller detail hereafter. The similarity with the female training does not go beyond these material offices, for the men are subjected in addition to an elaborate course of education by means of lectures and addresses delivered by the governor, and, we believe, on some themes, by the chaplain. These consist for the most part of a series of well-considered papers on the actual system within the walls, on the history and progress of prison administration, and giving some account of the labours of such friends of the hapless criminal as Howard, Buxton, and Elizabeth Fry. This is good, so far as it goes; but, in our opinion, the theoretical instruction might embrace a much wider range of subjects. It would be a valuable addition to the training of the warders that they should be thoroughly instructed, not only in the fundamental principles of judicial law, but in the complicated details which regulate the assigning of sentences to the various forms of crime, and the reasons which determine the nature and duration of such, in so far as they are legally fixed. The righteous administration of justice is a subject on which very conflicting opinions are held by every class of the community, and aspiring warders are not exempt from self-formed ideas on that vexed question which are likely to affect considerably their attitude towards evildoers, and which in their case, more than in almost any other, it would be advisable to bend in a right direction. We do not at all see why women should not have the benefit of lectures and addresses on these matters as well as men; but at present they are not so favoured. If prison administration is ever to become as just and efficacious as all would wish it to be, the systematic training of men and women for the position of prison officers should be enjoined upon every person, without exception, who aspires to such a post, and rigorously carried out as essential in all cases; in fact, no person, under any circumstances, should be placed in such a situation who has not gone through a complete and vigilant course of training.

There is, perhaps, no position in life which offers so much opportunity to men, "dressed in a little brief authority," to play those "tricks before high heaven" which Shakespeare tells us make the angels weep. As a rule, prison warders are chosen from the lower middle class—persons who have not been accustomed to command, who have never had servants or subordinates of any kind under their rule; and when they suddenly find themselves placed in authority over a mass of luckless individuals who are in absolute subjection to them, it acts as a species of intoxication in which the craving to use, and, it may be, abuse their sense of power is almost beyond their own control. It would be a very desirable improvement on the present custom if prison officers could be taken from a somewhat higher level, both as to social status and education, than they have been hitherto. When it happens that a man below even the usual standard succeeds in getting an appointment as warder, it is remarkable how quickly his undue satisfaction in the exercise of authority makes itself felt through the interior of the prison. The very tone of his voice has a ring that conveys a galling sense of humiliation when he gives the word of command to prisoners for the observance of such rules as that of standing with their faces turned to the wall, when a stranger, or even the well-known lady visitor, is passing them, and there are many ways in which he can make the temporary consciousness of superiority felt by men who outside the walls would be greatly his superiors in station. The secretary of the Howard Association, in his letter to the *Times*, quotes from Sir Edward du Cane—than whom no one has a wider knowledge of the subject or a better title to be heard on this point—as follows: "The officer who has charge of prisoners has such power for good or evil over his fellow-men that I do not think there are many positions more responsible than that which he occupies;" and an experienced chaplain, writing also on the subject, says: "The prison is first of all what the officers make it. Nothing in prison is worse for men than to come under an officer who has no moral interest in his work, no thorough confidence that prisoners can be helped to be better." While, therefore, on these general grounds we hope to see the day when the extremely inadequate number of thirty men and a much smaller contingent of women under training at present will give place to a definite and extensive arrangement for the training of all prison officers, we have to draw attention to other reasons which demand that it should not be only a certain favoured few who go through that necessary course, but that it should be absolutely obligatory upon all.

